# Association Mapping for Important Agronomic Traits in Safflower (*Carthamus tinctorius* L.) Core Collection Using Microsatellite Markers

**DOI:** 10.3389/fpls.2018.00402

**Published:** 2018-03-29

**Authors:** Heena Ambreen, Shivendra Kumar, Amar Kumar, Manu Agarwal, Arun Jagannath, Shailendra Goel

**Affiliations:** Department of Botany, University of Delhi, New Delhi, India

**Keywords:** safflower, association mapping, SSR markers, core collections, population structure, kinship analysis

## Abstract

*Carthamus tinctorius* L. (safflower) is an important oilseed crop producing seed oil rich in unsaturated fatty acids. Scarcity of identified marker-trait associations is a major limitation toward development of successful marker-assisted breeding programs in safflower. In the present study, a safflower panel (CartAP) comprising 124 accessions derived from two core collections was assayed for its suitability for association mapping. Genotyping of CartAP using microsatellite markers revealed significant genetic diversity indicated by Shannon information index (*H* = 0.7537) and Nei's expected heterozygosity (*I* = 0.4432). In Principal Coordinate Analysis, the CartAP accessions were distributed homogeneously in all quadrants indicating their diverse nature. Distance-based Neighbor Joining analysis did not delineate the CartAP accessions in consonance with their geographical origin. Bayesian analysis of population structure of CartAP demonstrated the unstructured nature of the association panel. Kinship analysis at population (*G*_*ij*_) and individual level (*F*_*ij*_) revealed absence of or weak relatedness between the CartAP accessions. The above parameters established the suitability of CartAP for association mapping. We performed association mapping using phenotypic data for eight traits of agronomic value (*viz*., seed oil content, oleic acid, linoleic acid, plant height, number of primary branches, number of capitula per plant, 100-seed weight and days to 50% flowering) available for two growing seasons (2011–2012 and 2012–2013) through General Linear Model and Mixed Linear Model. Our study identified ninety-six significant marker-trait associations (MTAs; *P* < 0.05) of which, several MTAs with correlation coefficient (*R*^2^) > 10% were consistently represented in both models and in both seasons for traits *viz*., oil content, oleic acid content, linoleic acid content and number of primary branches. Several MTAs with high *R*^2^-values were detected either in a majority or in some environments (models and/or seasons). Many MTAs were also common between traits (*viz*., oleic/linoleic acid content; plant height/days to 50% flowering; number of primary branches/number of capitula per plant) that showed positive or negative correlation in their phenotypic values. The marker-trait associations identified in this study will facilitate marker-assisted breeding and identification of genetic determinants of trait variability.

## Introduction

*Carthamus tinctorius* L., commonly known as “safflower,” contains seed oil with significantly high levels of nutritionally desirable unsaturated fatty acids, which is unique among oilseed crops (Fernandez-Martinez et al., 1993). Safflower is also used for extraction of dyes, several medicinal applications and as a plant factory for production of pharmaceuticals (Dajue and Mündel, 1996; Flider, 2013; Carlsson et al., 2014; Zhou et al., 2014). Additionally, its ability to grow under low moisture and high salinity conditions would give it a competitive edge over other oilseed crops in arid zones (Kaya et al., 2011; Bahrami et al., 2014; Yeilaghi et al., 2015; Ebrahimi et al., 2016). Currently, safflower is cultivated in around 20 countries in a total area of 1,140,002 hectares and production of 948,516 tons (FAOSTAT)^1^. The major producers of safflower are the Russian Federation (286,351 tons), Kazakhstan (167,243 tons), Mexico (121,767 tons), USA (99,830 tons), Turkey (58,000 tons), and India (53,000 tons) accounting for ~71% of total production (FAOSTAT)^1^. In spite of significant fluctuations in acreage under safflower cultivation, India was the highest average producer of the crop during 1994 to 2016. Nevertheless, safflower has not been able to create a niche for itself as a major oilseed crop. The primary factors that deter its cultivation are low yield and oil content, susceptibility to several biotic stresses, presence of spines, and lack of seed dormancy (Nimbkar, 2008). Genetic improvement of safflower is therefore, essential to increase its acceptability and utility as an oilseed crop of global importance.

A primary requisite for crop improvement is the presence of molecular and phenotypic diversity and identification of genes or quantitative trait loci (QTL) governing traits of agronomic importance. Genetic diversity in safflower has been analyzed using several molecular markers *viz*., Random Amplified Polymorphic DNA (RAPD), Inter Simple Sequence Repeat (ISSR), Amplified Fragment Length Polymorphism (AFLP), Simple Sequence Repeats (SSRs), and Single Nucleotide Polymorphism (SNPs) (Johnson et al., 2007; Yang et al., 2007; Amini et al., 2008; Khan et al., 2009; Sehgal et al., 2009; Chapman et al., 2010; Lee et al., 2014; Pearl and Burke, 2014; Ambreen et al., 2015; Kumar et al., 2015). These studies revealed high genetic differentiation among global safflower accessions and validated few “centers of similarity” of safflower that were identified based on morphological traits (Johnson et al., 2007; Chapman et al., 2010; Pearl and Burke, 2014; Kumar et al., 2015). Studies on development of linkage maps and tagging of agronomic traits using RAPDs, Restriction Fragment Length Polymorphism (RFLP), SSRs and SNP markers have been initiated in safflower (Hamdan et al., 2008, 2012; Mayerhofer et al., 2010; García-Moreno et al., 2011; Pearl et al., 2014). Linkage analysis and/or QTL mapping is a conventional method of identifying genomic regions governing simple and/or complex traits but requires development of bi-parental mapping populations, which is a time consuming process. Moreover, the allelic variation captured in QTL mapping is limited due to the use of bi-parental crosses and fewer recombination events are tested leading to low mapping resolution (Flint-Garcia et al., 2005). In contrast, association mapping (AM) is a faster and more efficient approach for high-resolution evaluation of complex traits and appears as a promising tool to circumvent limitations of linkage mapping (Yu and Buckler, 2006; Oraguzie and Wilcox, 2007; Abdurakhmonov and Abdukarimov, 2008). AM identifies relationships between traits and genetic polymorphisms in a heterogeneous assemblage of unrelated individuals using naturally occurring recombination events, thus enabling fine-scale mapping of traits. It has emerged as a useful approach for identifying marker-trait associations for several agronomic traits in various crop species (Abdurakhmonov and Abdukarimov, 2008; Zhu et al., 2008; Blair et al., 2009; Yang et al., 2010; Li et al., 2011; Upadhyaya et al., 2012, 2013; Xu et al., 2013; Zhang et al., 2014). Till date, Ebrahimi et al. (2017) is the only study that has explored marker-trait associations in safflower through association mapping using AFLP markers.

The choice of germplasm used for association mapping is critical and should incorporate a wide range of diversity capturing maximum number of historical recombination events (Flint-Garcia et al., 2005; Yan et al., 2011). Core collections have performed well as association mapping panels in various crop species (Blair et al., 2009; Li et al., 2011; Upadhyaya et al., 2013; Soto-Cerda et al., 2014; Zhang et al., 2014) since they are enriched with the maximum possible range of diversity (genetic and phenotypic) existing in the crop germplasm. Another advantage offered by core collections is that these collections often include unrelated individuals (accessions) which decreases the chances of identifying spurious marker-trait associations due to pre-existing population structure. In safflower, evaluation of global germplasm collections identified significant diversity for most of the desirable traits (oil content, fatty acid composition, resistance to biotic and abiotic stresses, and spines) (Ashri, 1975; Dwivedi et al., 2005; Kumar et al., 2016). To facilitate genetic dissection of complex traits, small, operational core collections have been developed in safflower (Johnson et al., 1993; Dwivedi et al., 2005; Kumar et al., 2016). These core collections can be effectively used for association mapping provided they demonstrate presence of high genetic variance (for better mapping resolution) and possess weak population structure and low kinship association among individuals (to avoid spurious associations between molecular markers and functional loci) during association analysis (Pritchard et al., 2000).

The present study describes evaluation of a safflower panel of 124 accessions derived from two core collections (reported earlier from our group; Kumar et al., 2016) for its suitability for association mapping and identification of SSR loci associated with eight traits of agronomic value (plant height, number of primary branches, number of capitula per plant, 100-seed weight, days to 50% flowering, seed oil content, oleic acid content and linoleic acid content) in the crop. The current work will facilitate mining of elite genes or loci for safflower breeding and conservation.

## Materials and methods

### Plant material and phenotypic evaluation

The present work utilize *C. tinctorius* L. accessions selected from two composite core collections of safflower, CartC1 (57 accessions) and CartC2 (106 accessions; Kumar et al., 2016). We merged accessions from these two core collections, removed redundancy (44 accessions were common to the two core collections) and included five Indian cultivars (Sharda, Manjira, Annigeri, PBNS-12 and TSF-1) assembling a final association panel with a non-redundant set of 124 accessions, which will hereafter be referred to as “CartAP” collection. Seed material of the accessions was obtained from USDA_ARS, WRPIS, Pullman, WA, USA while the Indian cultivars were procured from National Bureau of Plant Genetic Resources, New Delhi, India and University of Agricultural Sciences, Dharwad, India. Details of the plant material used including their plant identification (PI) number, geographical origin and information regarding their distribution in the two core collections are provided in Supplementary Table 1. Phenotypic evaluation of eight agronomic traits utilized in the present work *viz*., plant height, number of primary branches, number of capitula per plant, 100-seed weight, days to 50% flowering, seed oil content, oleic and linoleic acid was described by Kumar et al. (2016). The safflower accessions were evaluated in field conditions for two consecutive growing seasons (2011–2012 and 2012–2013) for the studied traits. The climatic variable data for the two growing seasons is provided in Supplementary Table 2.

### Isolation of genomic DNA and SSR genotyping

Genomic DNA was extracted from leaf tissues of 10 week-old seedlings of each accession as described by Ambreen et al. (2015). Ninety-three polymorphic SSR markers developed earlier in safflower (Ambreen et al., 2015) were used for genotyping the accessions. A three-primers PCR protocol (Perry, 2004) was followed for genotyping and the reaction mixture included 50 ng of template DNA, 1.5 mM MgCl_2_, 0.2 mM of each dNTPs, 0.05 μM IR700-labeled M13 primer, 0.05 μM M13-tailed forward primer, 0.05 μM reverse primer and 0.75 units of *Taq* DNA polymerase (Biotools, Spain). PCR amplifications were conducted on Veriti thermal cycler (Applied Biosystems, USA) under the following cycling conditions: initial denaturation at 96°C for 5 min followed by 28–30 cycles of 96°C for 45 s, appropriate annealing temperature (Ta°C, ranging from 55 to 65°C) for 30 s, extension at 72°C for 1 min and final extension at 72°C for 7 min. The IR700-labeled amplification products were size fractionated on 6.5% PAGE using 4,300 DNA analyzer system and analyzed following instructions provided in the user manual (LICOR, USA).

### Genetic analysis

Genetic diversity statistics including number of alleles (Na), major allele frequency (M_AF_), observed heterozygosity (*Ho*), expected heterozygosity (*He*) and degree of polymorphism generated by each marker (computed as polymorphism information content; PIC) were calculated using PowerMarker version 3.25 (Liu and Muse, 2005). Rare/private allelic richness per locus (*R*_*p*_) was determined based on rarefaction approach using HP-Rare v.1.0 (Kalinowski, 2005). Rarefaction allows evaluation of the allelic richness independent of the sample size (Kalinowski, 2004). Hardy Weinberg equilibrium and assessment of genetic diversity through expression of Shannon diversity index (*H*) and Nei's expected gene diversity (*I*) was tested using POPGENE version 1.32 (Yeh et al., 1999). Information about the genetic location of these SSR markers on safflower linkage groups was derived from Bowers et al. (2016) through BLASTN search using standalone Blast+ version 2.2.26 (ftp://ftp.ncbi.nlm.nih.gov/blast/executables/blast+/2.2.26/). The genomic sequences of 93 SSR loci were used as query for search against the genetic map assembled into twelve linkage groups of safflower by Bowers et al. (2016).

### Distance-based genetic analysis

Genetic relationships between sampled accessions were elucidated through construction of an unrooted Neighbor Joining (NJ) dendrogram based on simple matching coefficient using Darwin version 6.0 (Perrier and Jacquemoud-Collet, 2006). A bootstrap value of 1,000 replicates was used to test the reliability of the NJ dendrogram. Principal Coordinate Analysis (PCoA) was performed using Darwin version 6.0.

### Analysis of population structure using bayesian method

The population genetic structure was studied using Bayesian clustering method implemented in STRUCTURE version 2.3.4 (Pritchard et al., 2000). An admixture model and correlated allele frequencies were chosen for estimating proportion of ancestral contribution in each accession. We tested various *K*-values ranging from 1 to 25 with 10 independent replications at each *K*, 100,000 generations burnin period and 200,000 Markov Chain Monte Carlo (MCMC) repetitions. The analysis was performed independent of the geographical origin of accessions. The optimal *K*-value for the dataset was obtained by assessment of the results using STRUCTURE HARVESTOR (Earl, 2012). Accessions with membership proportions (*Q*-value) >80% were considered as pure and part of their corresponding cluster while accessions with membership proportions lesser than 80% were adjudged as admixtures. Calculation of pairwise F_ST_ and Analysis of molecular variance (AMOVA) between the sub-populations of STRUCTURE were performed using GenAlEx version 6.5 (Peakall and Smouse, 2012) with 1,000 permutations.

### Kinship coefficient

Relatedness between individuals was estimated through computation of kinship coefficients (*F*_*ij*_; individual level) following Loiselle et al. (1995). *F*_*ij*_ measures the relative probabilities of identity in descent for random genes between two individuals, *i* and *j*. Kinship coefficient matrix between all pairs of accessions of the association panel was generated using SPAGeDi v 1.5 (Hardy and Vekemans, 2002). All the negative *F*_*ij*_*-*values were treated as zero (Hardy and Vekemans, 2002; Yu et al., 2006). The same program was used for evaluation of average kinship coefficient (*G*_*ij*_; population level) between sub-populations inferred using STRUCTURE.

### Association mapping

Association mapping was performed for eight quantitative traits *viz*., seed oil content, oleic acid content, linoleic acid content, 100-seed weight, plant height, number of primary branches, days to 50% flowering and number of capitula per plant for the two seasons independently. Only SSR markers with known positions on linkage groups were used for association mapping. Both General linear model (GLM) and Mixed linear model (MLM) were applied for assessment of marker-trait associations (MTA) in TASSEL v 5.0 (Bradbury et al., 2007) following the user manual. The GLM model includes population structure (Q matrix) but does not include kinship relationships (K matrix) and can often identify false positives in association mapping. To overcome this limitation, the MLM was proposed which incorporates both population structure (Q) and kinship (K) information to decrease spurious associations (Yu et al., 2006). The association between marker and trait was considered significant at a *P* < 0.05. The phenotypic variation explained by each marker-trait association was studied through correlation coefficient (*R*^2^).

## Results

### SSR-based genotyping and statistical analysis

Genotyping of 124 safflower accessions of the CartAP collection was performed using 93 polymorphic SSR markers. The SSR markers generated a total of 311 alleles with an average of 3.34 alleles per locus. The number of alleles per locus ranged from 2 to 8 (Supplementary Table 3). The highest number of alleles (8) was detected for loci NGSaf_265 and NGSaf_282 (Table 1). The major allele frequency (M_AF_) was high and varied from 0.342 to 0.97 with an average of 0.66 (Supplementary Table 3). The observed heterozygosity (*Ho*) had a low average value of 0.112 and ranged from 0 to 0.96 (Supplementary Table 3). The gene diversity or expected heterozygosity (*He*) of studied markers ranged from 0.016 for NGSaf_117 to 0.76 for NGSaf_281 with an average of 0.438 (Supplementary Table 3). Information on genetic variability at the 93 SSR loci among accessions of the CartAP collection is summarized in Table 1.

**Table 1:** Summary statistics for genetic variability at 93 SSR loci.

**MARKER PARAMETERS**	
Number of accessions sampled	124
**ALLELES**	
Total number of alleles	311
Average number of alleles per locus	3.34
Range of alleles	2–8
SSR loci with highest number of alleles^#^	NGSaf_265 and NGSaf_282 (8 alleles)
***Ho***	
Average *Ho*	0.112
SSR locus with highest *Ho*^#^	NGSaf_294 (0.958)
SSR locus with lowest *Ho*^#^	NGSaf_14, NGSaf_45, NGSaf_63, NGSaf_98, NGSaf_114, NGSaf_117, NGSaf_130, NGSaf_145, NGSaf_151, NGSaf_154, NGSaf_173, NGSaf_248 (0)
***He***	
Average *He*	0.438
SSR locus with highest *He*^#^	NGSaf_281 (0.76)
SSR locus with lowest *He*^#^	NGSaf_117 (0.016)
**PIC**	
Average PIC-value	0.38
SSR locus with highest PIC-value^#^	NGSaf_281 (0.73)
SSR locus with lowest PIC-value^#^	NGSaf_117 (0.02)

*He, gene diversity or expected heterozygosity; Ho, observed heterozygosity; PIC, polymorphic information content*,

# *Values for each parameter are provided in parenthesis*.

The relative informativeness of these markers was estimated by measurement of polymorphic information content (PIC) for each locus. The PIC-value averaged at 0.38 and ranged from 0.02 for NGSaf_117 to 0.73 for NGSaf_281. Nineteen microsatellite loci were highly polymorphic with PIC > 0.5 and ranged from 0.502 to 0.736 (Supplementary Table 3). A major proportion of microsatellite loci (63) were moderately polymorphic with PIC-values between 0.25 to 0.5 while 12 loci generated low polymorphism with PIC < 0.25. Based on marker statistics, the NGSaf_281 locus was most informative and had highest utility value among the 93 SSR loci. We were able to assign map positions to 48 out of the 93 SSR loci based on the genetic map of safflower reported by Bowers et al. (2016). SSR locus specific details (Na, M_AF_, *He, Ho*, and PIC) and their map positions are provided in Supplementary Table 3. Mean allelic richness and private allelic richness (number of unique alleles) were measured based on rarefaction approach implemented in HP-rare v 1.0. The mean allelic richness per locus was estimated as 1.287 while private allelic richness was 0.0186. At a significance level of *P* < 0.05, all loci except NGSaf_300 deviated from Hardy-Weinberg equilibrium (HWE) while at *P* < 0.01, all loci deviated from HWE (Supplementary Table 3).

### Genetic diversity

High genetic differentiation was observed among CartAP accessions based on the genetic diversity indices—Shannon information index (*H*; 0.7537) and Nei's expected heterozygosity (*I*; 0.4432). Genetic diversity in the 10 proposed regional gene pools and regions of secondary introduction of safflower (America and Australia) were also evaluated (Table 2). Among the regional gene pools, the Shannon information index varied from 0.4158 to 0.7211 while Nei's expected heterozygosity ranged from 0.2595 to 0.4265. Based on diversity indices, accessions from the Indian subcontinent and American region were the most diverse with an estimated Shannon information index and Nei's expected heterozygosity value of *H* = 0.7028 and 0.7211 and *I* = 0.4265 and 0.4227, respectively. Near East (*H* = 0.4158; *I* = 0.2595) and Turkey (*H* = 0.4650; *I* = 0.2981) were genetically least diverse based on our assessment using SSR markers. Within-region segregation assessed using SSR markers revealed the highest number of polymorphic loci among accessions from the Indian subcontinent (91) followed by America (90) and Iran-Afghanistan (85). The least number of polymorphic loci were recorded for accessions from Near East (60) and Turkey (60; Table 2).

**Table 2:** Genetic diversity indices of CartAP accessions based on their regional gene pool distribution.

**Regional gene pool**	***N***	**P_l_**	**PP_l_ (%)**	***H***	***I***
Far East	15	84	90.3	0.6029	0.3782
Indian subcontinent	32	91	97.8	0.7028	0.4265
Iran-Afghanistan	12	85	91.4	0.6016	0.3596
Egypt	5	72	77.4	0.5289	0.3449
Europe	9	84	90.3	0.5966	0.3818
Near East	6	60	64.5	0.4158	0.2595
Turkey	5	60	64.5	0.4650	0.2981
America	30	90	96.8	0.7211	0.4227

*N, Number of accessions; P_l_, Number of polymorphic loci; PP_l_, Percent polymorphism; H, Shannon information index; I, Nei's gene diversity*.

### Genetic relationships and population structure

#### Neighbor joining (NJ) dendrogram and principal coordinate analysis (PCoA)

**Figure 1:**
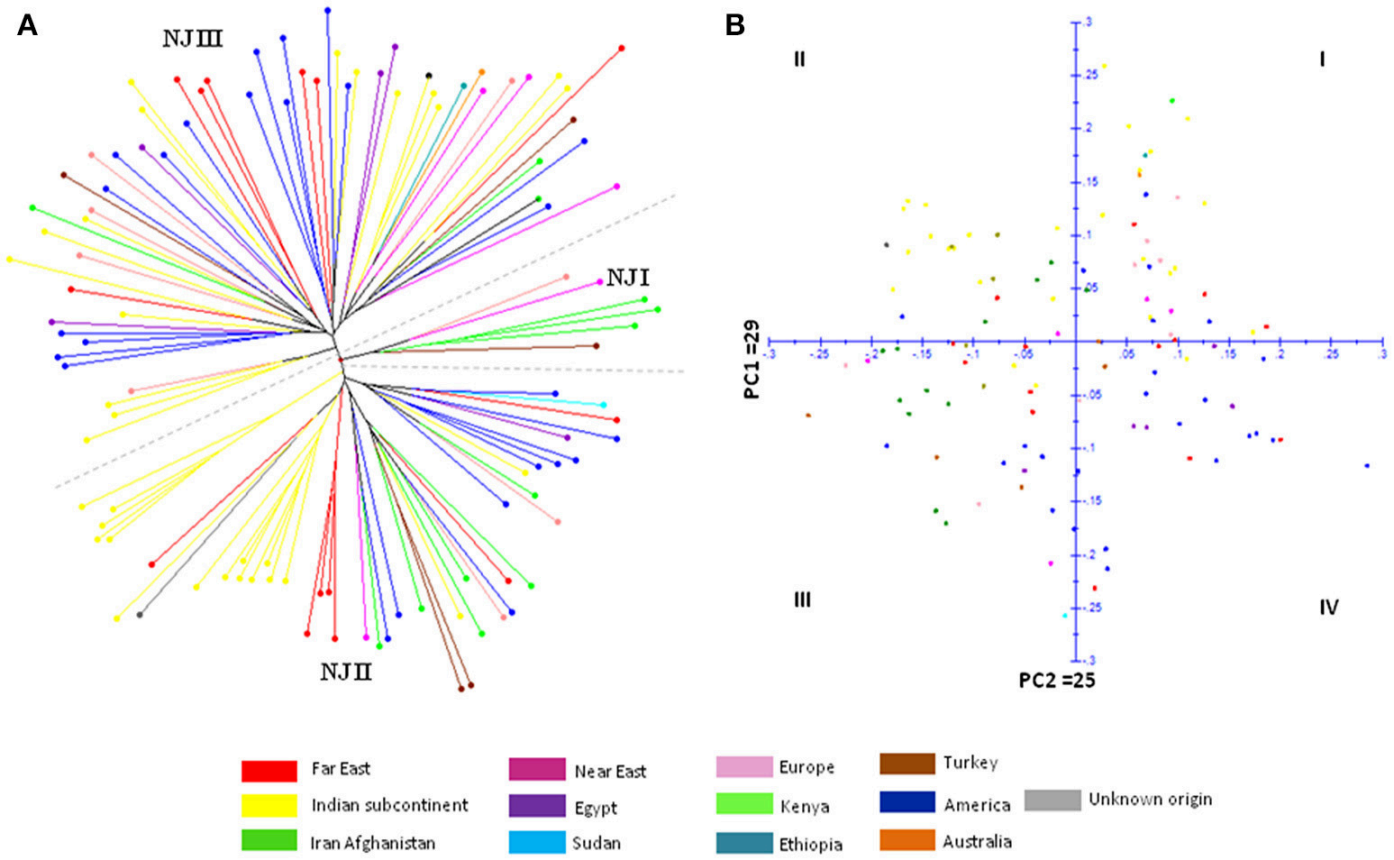
**(A)** Neighbor Joining dendrogram elucidating the genetic relationships between 124 CartAP accessions using 93 SSR loci based on simple matching coefficient. NJ clusters (NJ I–NJ III) are demarcated through dashed lines. **(B)** Scatter plot for Principal Coordinate Analysis (PCoA) exhibiting the distribution of CartAP accessions on two main axes. Primary axes 1 and 2 captured 29 and 25% of the total variance, respectively. Color codes used for different regional pools are provided.

The genetic relatedness between safflower accessions of CartAP was analyzed by construction of Neighbor-Joining (NJ) dendrogram based on simple matching coefficient (Figure 1A). The 124 accessions were grouped into three major clusters (named as NJ I–NJ III) with 7, 48, and 69 accessions, respectively. However, no distinction could be drawn among these groups based on their geographical origin with each of the major cluster exhibiting heterogeneous clustering of accessions (Figure 1A). We also obtained low bootstrap support (< 50%) for majority of the nodes of the NJ dendrogram and only very few internal nodes exhibited high bootstrap value > 50%. All the five Indian cultivars analyzed in the present study clustered together in NJ II indicating their narrow genetic base. Distance-based NJ analysis indicated lack of genetic relationships among these accessions. In Principal Coordinate Analysis (PCoA) based on SSR data, the principal axes 1 and 2 explained 29 and 25% of the total molecular variance, respectively (Figure 1B). In agreement with the clustering pattern of NJ dendrogram, aggregation of accessions into discrete groups relative to their geographical origin was not observed in the PCoA plot. The CartAP accessions tend to disperse homogeneously in all the four quadrants indicating the diverse nature of sampled accessions (Figure 1B).

#### Population structure and differentiation

**Figure 2:**
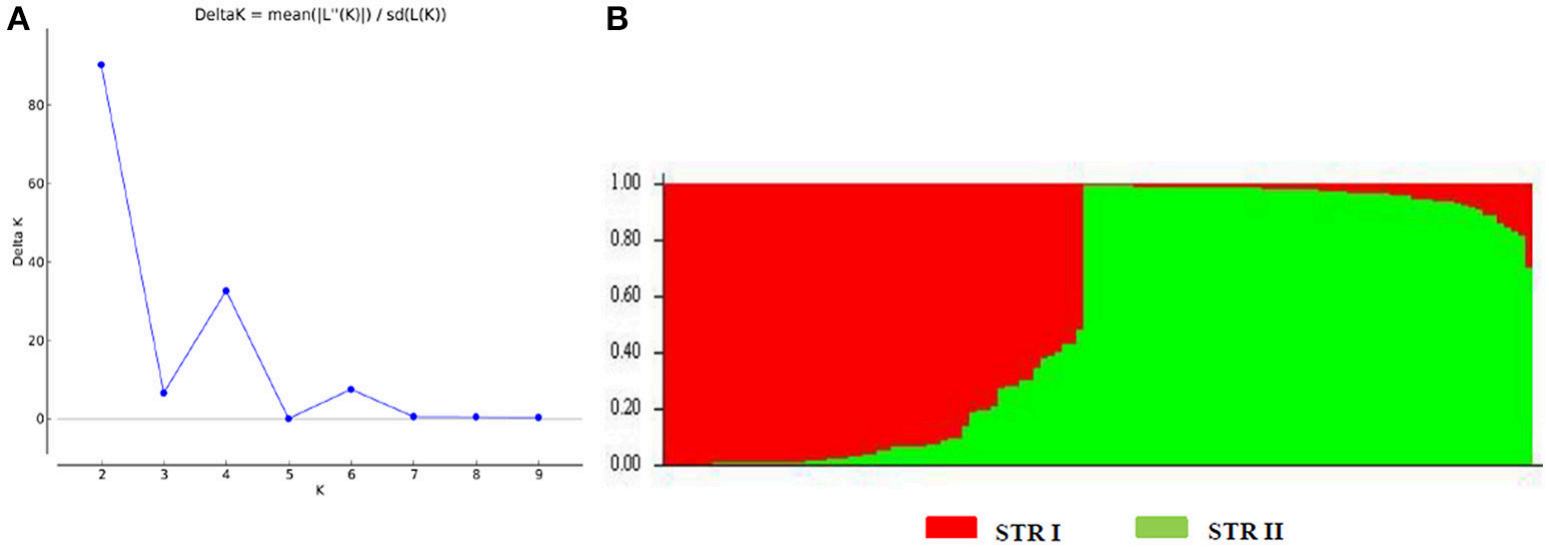
Population structure of CartAP collection inferred using Bayesian-based program STRUCTURE. **(A)** Estimation of hypothetical sub-populations using Δ*K-*values. The number of identified sub-populations were two (*K* = 2). **(B)** Population structure analysis of CartAP accessions at *K* = 2 based on inferred ancestry (Q matrix). The sub-populations were named STR I and STR II.

**Figure 3:**
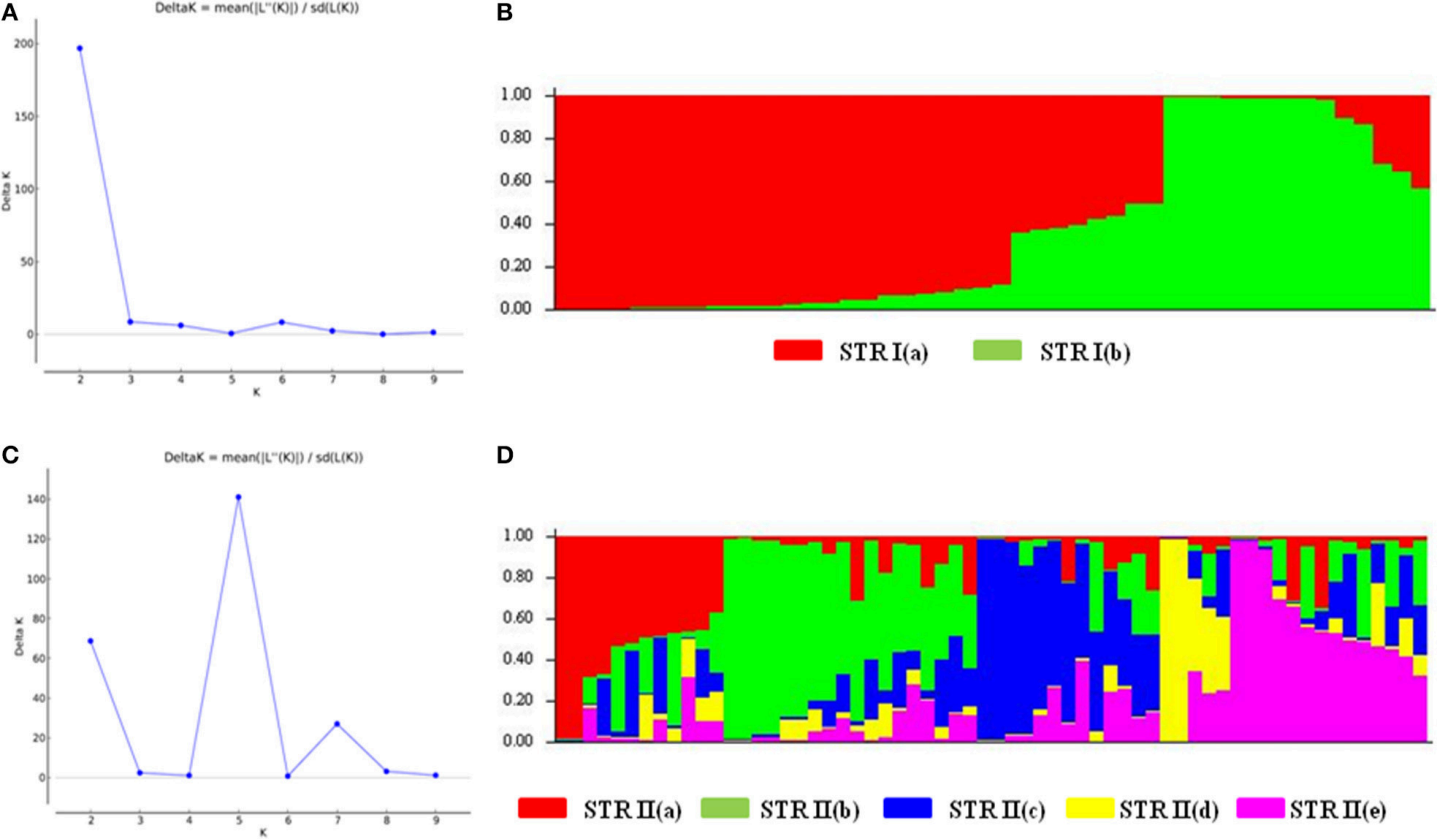
Hierarchical population structure analysis **(A)** Estimation of sub-population for STR I. Maximum number of sub-populations were inferred at *K* = 2. **(B)** Population structure for STR I at *K* = 2 based on Q matrix. The sub-populations were designated as STR I(a) and STR I(b) **(C)** Estimation of sub-populations for STR II. Maximum number of subpopulations were inferred at *K* = 5. **(D)** Population structure for STR II at *K* = 5 based on Q matrix. The sub-populations were named as STR II(a)–STR II(e). Color codes for each sub-population are provided.

We also analyzed the underlying population structure of CartAP through Bayesian-based approach using STRUCTURE v 2.3.4. Log mean probability and change in log probability (Δ*K*) were determined using STRUCTURE HARVESTER, which generated the highest peak at *K* = 2 (Figure 2A). Based on membership proportions, safflower accessions with ≥ 80% of shared ancestry were delineated into two major clusters (STR I and STR II) with 46 and 62 accessions, respectively (Figure 2B). Each of these clusters included diverse accessions originating from different regional pools. Sixteen accessions with < 80% of shared ancestry were categorized as admixtures. Additional smaller peaks observed at *K* = 4 and *K* = 6 implied presence of sub-groups within the two major groups (Figure 2A). Therefore, an independent STRUCTURE run was performed for STR I and STR II with *K*-values ranging from 1 to 10. Sub-clustering of STR I identified the highest peak at *K* = 2 (Figure 3A). The two sub-clusters designated as STR I(a) and STR I(b), contained 32 and 14 accessions, respectively (Figure 3B). Sub-clustering of the second major group, STR II yielded highest peak at *K* = 5 (Figure 3C). The five sub-clusters were designated as STR II(a), STR II(b), STR II(c), STR II(d), and STR II(e) and contained 12, 18, 13, 5, and 14 accessions, respectively (Figure 3D). Clustering of accessions in STRUCTURE analysis was heterogeneous and in concordance with the results of NJ and PCoA. The regional distribution of accessions in the seven sub-populations is given in Table 3. Among the sub-populations, a substantial number of accessions were admixtures (Figures 3B,D).

**Table 3:** Distribution of CartAP accessions in different sub-populations derived from STRUCTURE analysis.

**Regional gene pools**	**Number of accessions**	**STRI**		**STRII**					**Admixtures**
		**STRI(a)**	**STRI(b)**	**STRII(a)**	**STRII(b)**	**STRII(c)**	**STRII(d)**	**STRII(e)**	
Far East (FE)	15	3	–	2	5	–	–	1	4
Indian subcontinent (IS)	32	5	12	2	1	8	1	3	4
Iran-Afghanistan (IA)	12	9	–	–	–	–	–	3	1
Near East (NE)	6	2	1	–	–	1	–	2	–
Turkey (TU)	5	3	–	–	–	–	–	1	1
Egypt (EG)	5	1	–	2	2	–	–	–	–
Sudan (SU)	1	1	–	–	–	–	–	–	–
Kenya (KE)	1	–	–	–	–	1	–	–	–
Ethiopia (ET)	1	-	–	–	–	1	–	–	–
Europe (EU)	9	2	–	2	1	–	3	1	–
America (US)	30	6	–	4	9	1	1	3	6
Australia (AUS)	1	–	–	–	–	1	–	–	–
Unknown origin (UN)	1	–	1	–	–	–	–	–	–
Total	124	32	14	12	18	13	5	14	16

**Figure 4:**
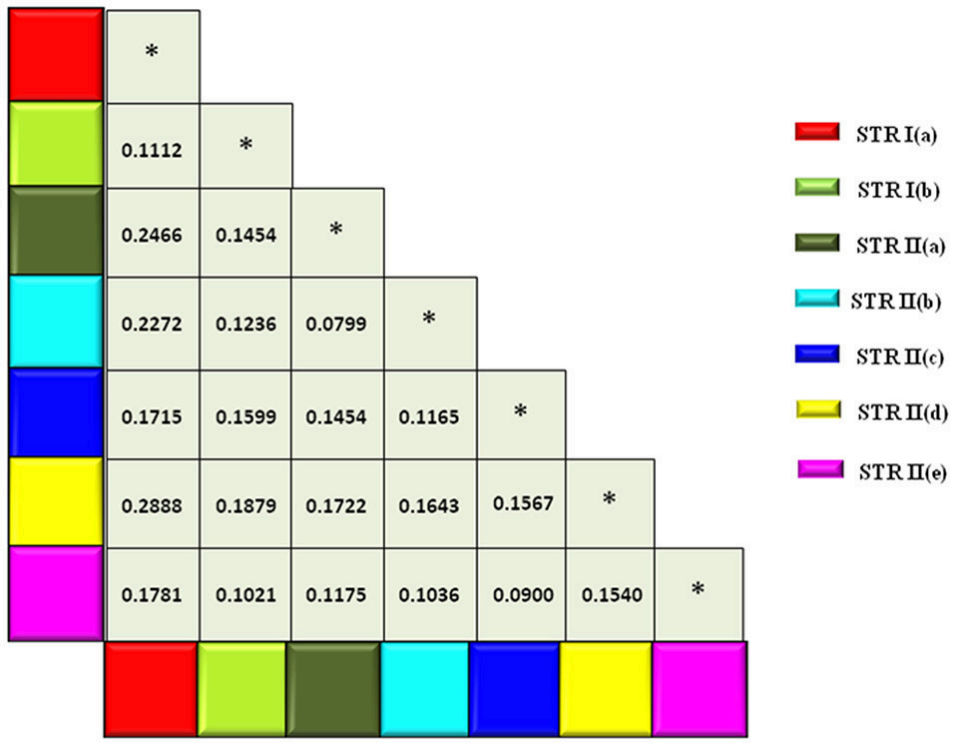
Matrix showing pairwise F_ST_-values between sub-populations inferred through hierarchical STRUCTURE analysis. Color codes are provided for each sub-population. ^*^Denotes F_ST_-values between same sub-population.

The expected heterozygosity for the seven sub-clusters varied from 0.1726 for STR II(d) to 0.4352 for STR I(a) (Table 4). The genetic divergence among the seven sub-populations was measured through pair-wise F_ST_, which ranged from 0.0799 for STR II(b) and STR II(a) to 0.2888 for STR II(d) and STR I(a) (*P* < 0.001; Figure 4). A hierarchical AMOVA analysis was performed among and within regional pools of CartAP accessions, which provided an estimated molecular variance of 7 and 93%, respectively (Table 5). Similar evaluation was conducted for seven sub-clusters derived through STRUCTURE analysis of safflower accessions. It provided a molecular variance of 16 and 84% among and within sub-clusters, respectively (Table 5). Genetic differentiation among the sub-clusters was highly significant (*P* < 0.0001).

**Table 4:** Expected heterozygosity of the sub-populations derived through STRUCTURE analysis.

**Sub-clusters**	**Expected heterozygosity**
STR I	0.469
STR I(a)	0.4352
STR I(b)	0.2838
STR II	0.4217
STR II(a)	0.2230
STR II(b)	0.3475
STR II(c)	0.2669
STR II(d)	0.1726
STR II(e)	0.2845

**Table 5:** Analysis of molecular variance (AMOVA) between and within regional gene pools and between and within sub-populations derived through hierarchical STRUCTURE analysis.

	Est. Var.^*^	**Percent of variance**	***P*-value^a^**
**REGIONAL GENE POOLS**			
Between populations	1.413	7	0.001
Within populations	19.024	93	0.001
Total	20.437	100	
**SEVEN SUB-POPULATIONS DERIVED FROM STRUCTURE**			
Between populations	3.367	16	0.001
Within populations	17.524	84	0.001
Total	20.891	100	

* *Estimated variance*.

a *With 999 data permutations*.

### Kinship analysis at individual and population level

**Figure 5:**
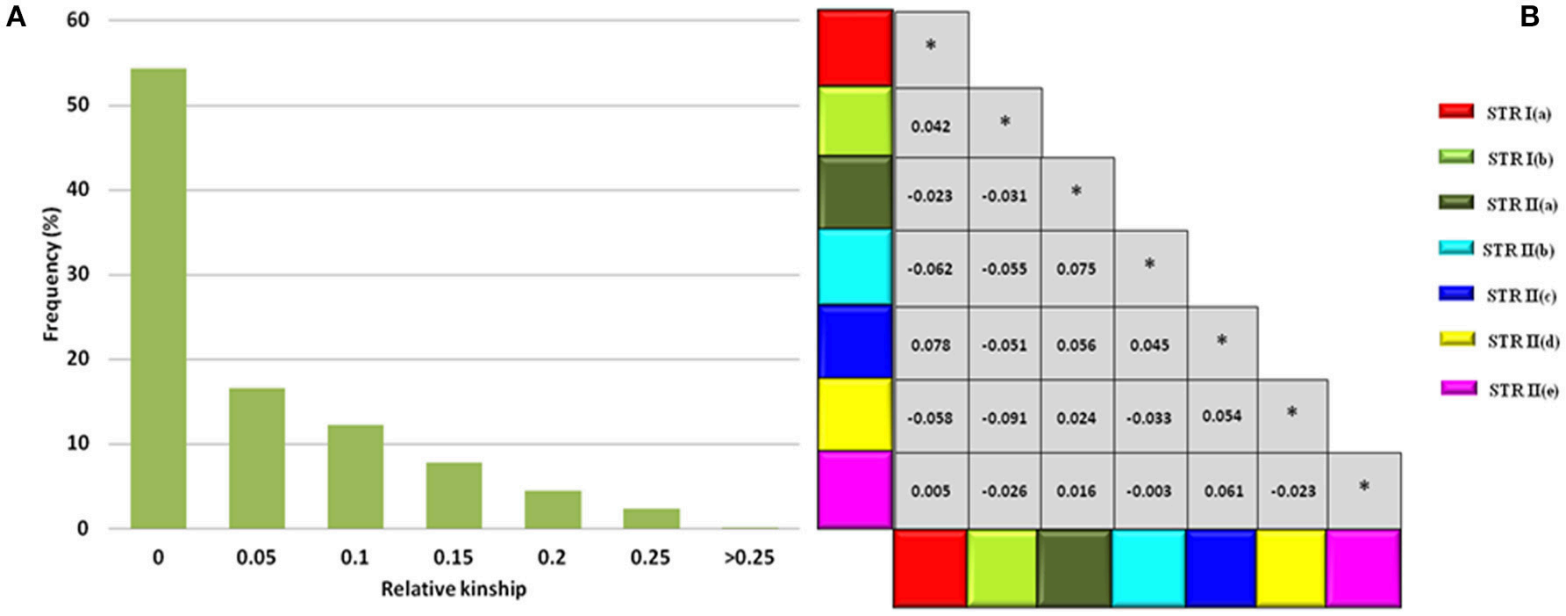
Kinship estimates at individual and population level. **(A)** Frequency distribution for global pairwise kinship estimates (*F*_*ij*_) for CartAP accessions. **(B)** Matrix showing pairwise *G*_*ij*_*-*values between sub-populations inferred through hierarchical STRUCTURE analysis. Color codes are provided for each sub-population. ^*^Denotes *G*_*ij*_*-*values values between same sub-population.

Based on 93 SSR markers, the co-ancestry or kinship coefficient (*F*_*ij*_) between any two safflower accessions of CartAP averaged at a value of 0.043. A major proportion of the pairs of safflower accessions (~54.3%) had zero pairwise kinship estimates followed by 16.59% pairs having kinship estimates between 0.001 to 0.05, 12.24% pairs between 0.051 to 0.1, 7.8% pairs between 0.11 to 0.15, 4.5% pairs between 0.151 to 0.20 and 2.4% pairs between 0.201 to 0.25 (Figure 5A). A minimal fraction of safflower accession pairs (0.022%) exhibited kinship estimate >0.25. At the population level, *G*_*ij*_-values were estimated to assess kinship associations between the seven inferred sub-populations derived from STRUCTURE analysis. The mean kinship coefficients (*G*_*ij*_) ranged from 0 to 0.0783 (Figure 5B) between the sub-populations. Negative values of *G*_*ij*_ were treated as zero for the current analysis. The kinship analysis indicated that most CartAP accessions and the inferred sub-populations exhibited no or weak level of genetic relatedness at the individual and population level, respectively.

### Association mapping

The 48 mapped SSR markers were distributed across all the 12 linkage groups of safflower (Supplementary Table 3) with maximum number of SSR loci (9) on linkage group (LG) 6. Identification of markers linked with each of the eight agronomic traits of safflower (seed oil content, oleic acid content, linoleic acid content, 100-seed weight, plant height, number of primary branches, days to 50% flowering and number of capitula per plant) was done through association analysis and performed independently for the two growing seasons. The range and mean values of the eight phenotypic traits evaluated for CartAP accessions are provided in Table 6. Association mapping using GLM and MLM models identified 96 significant marker-trait associations (*P* < 0.05) for the eight traits across two growing seasons (2011–2012 and 2012–2013). Twenty-eight out of 48 SSR loci accounted for these 96 significant associations. A comparatively higher number of significant marker-trait associations were observed through GLM (60) than MLM (36). Out of the 96 significant marker-trait associations (MTAs) detected based on *P*-value, 63 associations exhibited correlation value (*R*^2^) ≥ 10%. Those MTAs, which were detected in more than one season/model and exhibited *R*^2^ ≥ 10% in at least one model and/or season were retained for further analysis (Table 7). As an exception, we have included the MTA for NGSaf_279 with number of capitula per plant, since it was consistently detected in all the seasons and models with *R*^2^ ranging from 7.4 to 9.3%.

**Table 6:** Mean and range of different phenotypic traits for CartAP accessions.

**Phenotypic traits**	**Mean**	**Range**
Oil content (%)	32	16–50
Oleic acid (%)	27	10–79
Linoleic acid (%)	65	13–87
100-seed weight (gm)	4	1–8
Plant height (cm)	156	94–226
Number of capitula per plant	82	16–203
Number of primary branches	16	6–33
Days to 50% flowering	141	119–160

**Table 7:** Marker-trait associations identified through General linear model (GLM) and Mixed linear model (MLM) using phenotypic data of season 2011–2012 and 2012–2013.

**Traits**	**SSR marker ID**	**LG^@^**	**SEASON 2011–2012**				**SEASON 2012–2013**			
			**GLM**		**MLM**		**GLM**		**MLM**	
			***R*^2^ (%)**	***P*-value**	***R*^2^ (%)**	***P*-value**	***R*^2^ (%)**	***P*-value**	***R*^2^ (%)**	***P*-value**
**OIL CONTENT**										
	NGSaf_15	3	22.3	1.363E-5	10.4	0.0436	23.4	6.85E-06	16	0.004
	NGSaf_148	7	–	–	–	–	11.5	0.02	–	–
	NGSaf_201	11	12.9	0.011	–	–	–	–	–	–
	NGSaf_255	1	9.7	0.033	–	–	16	0.001	–	–
	NGSaf_300	12	16.7	6.026E-4	16.2	0.016	17.9	2.99E-04	13.8	0.042
**OLEIC ACID**										
	NGSaf_67	7	11.4	.005	11.4	0.017	11.6	0.004	11.6	0.016
	NGSaf_210	4	–	–	14.3	0.031	–	–	14.6	0.028
	NGSaf_309	5	–	–	34.1	.002	–	–	34	0.002
**LINOLEIC ACID**										
	NGSaf_67	7	10.9	0.006	10.9	0.021	–	–	16.7	0.039
	NGSaf_83	3	–	–	–	–	12.4	0.014	–	–
	NGSaf_155	10	–	–	–	–	19.1	0.041	–	–
	NGSaf_210	4	14.3	0.013	14.4	0.031	14	0.007	17.9	0.004
	NGSaf_309	5	–	–	34.1	0.002	–	–	–	–
**100-SEED WEIGHT**										
	NGSaf_101	4	–	–	–	–	8.9	0.006	15.3	0.034
	NGSaf_306	6	14.7	0.031	24.52	0.011	13	0.034	–	–
	NGSaf_309	5	24.1	4.2291E-4	22.9	0.044	15.5	0.022	–	–
**PLANT HEIGHT**										
	NGSaf_23	6	10	0.009	–	–	–	–	–	–
	NGSaf_83	3	12.5	0.013	–	–	–	–	–	–
	NGSaf_101	4	14.1	0.019	–	–	12.2	0.049	–	–
	NGSaf_156	6	11.8	0.023	–	–	13.7	0.01	13.9	0.033
	NGSaf_173	5	10.6	3.84E-4	4.4	0.027	–	–		
	NGSaf_296	6	14.7	0.007	–	–	12.7	0.021	13.1	0.018
**NUMBER OF CAPITULA PER PLANT**										
	NGSaf_279	6	7.4	0.037	9.3	0.037	8.1	0.024	8.5	0.051
	NGSaf_309	5	15.4	0.023	25.7	0.023	–	–	–	–
**NUMBER OF PRIMARY BRANCHES**										
	NGSaf_15	3	–	–	–	–	11.8	0.008	12.4	0.021
	NGSaf_83	3	13.3	0.009	–	–	10.5	0.037	10.5	0.047
	NGSaf_279	6	8.9	0.015	10.8	0.018	8	0.024	8.9	0.043
**DAYS TO 50% FLOWERING**										
	NGSaf_92	9	–	–	11.4	0.014	7.9	0.026	11.6	0.014
	NGSaf_101	4	12.6	0.037	–	–	–	–	–	–
	NGSaf_201	11	11.9	0.017	11.4	0.014	11.8	0.019	–	–
	NGSaf_255	1	10.7	0.021	–	–	–	–	–	–
	NGSaf_296	6	–	–	–	–	11.1	0.043	–	–

@ *Linkage group; R^2^, correlation coefficient*.

For the 2011–2012 season, 19 and 14 significant MTAs with *R*^2^ ≥ 10% were detected using GLM and MLM analysis, respectively (Table 7). Twelve MTAs were common between both the models for the studied traits. In GLM, the least number of significant associations (*R*^2^ ≥ 10%) were recorded for oleic acid, number of capitula per plant and number of primary branches (1) while the maximum number of MTAs (6) was recorded for plant height. The correlation value (*R*^2^) for these MTA ranged from 10 to 24.1%. The MTA between 100-seed weight and NGSaf_306 displayed the highest *R*^2^ (24.1%; Table 7). In MLM analysis, the highest number of significant loci was associated with oleic acid and linoleic acid (3) while the least number (1) of MTA were detected for number of capitula per plant and number of primary branches. MLM analysis could not identify any MTA with *R*^2^ ≥ 10% for plant height. Among the MTA detected through MLM, the *R*^2^-values ranged from 10.4 to 34.1%. MTA between oleic/linoleic acid and NGSaf_309 displayed the highest *R*^2^ (34%).

For the 2012–2013 season, we detected 17 and 13 significant MTAs with *R*^2^ ≥ 10% for the eight agronomic traits using GLM and MLM analysis, respectively (Table 7). Twelve MTAs were common between both the models for the studied traits. In GLM, the number of significant SSR loci associated with each trait ranged from 1 (for oleic acid) to 4 (for oil content) with *R*^2^-value ranging from 10.5 to 23.4%. The MTA between oil content and NGSaf_15 had the highest *R*^2^-value of 23.4% (Table 7). In the MLM model, the least number of loci (1) were observed for 100-seed weight and days to 50% flowering while maximum number of loci (3) was found to be linked with oleic acid. These 13 MTAs had *R*^2^-values ranging from 10.5 to 34.1%. Among these MTAs, we observed the highest *R*^2^-value of 34% between oleic acid and NGSaf_309.

A cumulative assessment of MTAs for each trait identified several significant (*P* < 0.05; *R*^2^ > 10) associations, which were consistently represented in both models and in both seasons. For example, NGSaf_15 and NGSaf_300 for oil content, NGSaf_67 for oleic acid content, NGSaf_210 for linoleic acid content, NGSaf_279 for number of primary branches and number of capitula per plant were detected (Table 7). We also observed MTAs with high correlation values, which were detected either in a majority or only in some environments (models and/or seasons). Many MTAs were also common between traits that showed positive or negative correlation in their phenotypic values (Table 7).

## Discussion

Our earlier work on analysis of a representative global germplasm collection of ~531 safflower accessions identified significant genetic and phenotypic variability in the crop (Kumar et al., 2015, 2016) that could be effectively used to improve traits of agronomic value to enhance crop yield and productivity. Since identification of desirable alleles from large germplasm collections is tedious and time-consuming, core collections encompassing the prevailing genetic and phenotypic diversity of the crop are often developed. In safflower, three studies have reported development of core collections (Johnson et al., 1993; Dwivedi et al., 2005; Kumar et al., 2016). Core collections described by Johnson et al. (1993) and Dwivedi et al. (2005) were based only on morphological and geographical parameters and did not include information on molecular diversity of the crop. They were also comparatively larger with 210 and 570 accessions, respectively. In contrast, two core collections, CartC1 and CartC2, described by Kumar et al. (2016) were constructed based on Maximization (M) strategy using genetic (AFLP), phenotypic and geographical data and were much smaller (57 and 106 accessions, respectively). Additionally, CartC1 and CartC2 include accessions from eight and ten regional gene pools of safflower (Ashri, 1975), respectively and also have representation from secondary regions of introduction. The present study analyzed the suitability of the above core collections (CartC1 and CartC2) for association mapping through assessment of its genetic diversity and population structure using SSR markers followed by establishment of marker-trait associations for eight agronomically important traits.

### CartAP collection is an effective panel for association mapping

#### High genetic variation in CartAP accessions

SSR markers have been extensively used for assessment of association mapping panel and marker-trait associations (Breseghello and Sorrells, 2006; Agrama et al., 2007; Jun et al., 2008; Yang et al., 2010; Rezaeizad et al., 2011). Being co-dominant markers, they are considered more robust for estimation of population structure and kinship relatedness than dominant markers (Zhu et al., 2008). A large proportion of our tested microsatellite loci (88%) generated moderate to high level of polymorphism, which indicates their efficiency in differentiating *C. tinctorius* L. accessions of CartAP. The mean PIC-value (0.38) obtained in our study using SSR markers on the CartAP collection corroborated well with previous SSR-based studies in safflower (Table 8; Hamdan et al., 2011; Barati and Arzani, 2012; Derakhshan et al., 2014; Lee et al., 2014). The allelic range and average number of alleles per locus observed in the present study were lower than those observed by Chapman et al. (2009; Table 8) probably due to the inclusion of wild species in their study. High genetic variability among CartAP accessions are also illustrated by the diversity indices, *H* = 0.7537 and *I* = 0.4432. This wide genetic diversity is particularly advantageous for association studies as it offers greater allelic diversity with inclusion of rare variants and higher mapping resolution due to representation of historical recombination events from a global population. Thus, the CartAP panel fulfilled the prerequisite of a highly differentiated panel for association mapping (Flint-Garcia et al., 2005).

**Table 8:** Comparative account of SSR marker statistics of the present and previous studies in safflower.

	**Present study**	**(Chapman et al., 2009)**	**(Hamdan et al., 2011)**	**(Barati and Arzani, 2012)**	**(Lee et al., 2014)**	**(Derakhshan et al., 2014)**
Number of accessions sampled	124	27	10	48	100	42
Number of polymorphic SSR markers	93	104	64	42	30	33
Allelic range	2–8	2–15	2–8	2–8	2–7	2–8
Average number of alleles per locus	3.3	6	3.2	3.4	2.8	3.8
Mean expected heterozygosity (*He*)	0.438	0.54	0.52	0.37	0.386	^*^
PIC	0.38	0.32	^*^	0.32	0.325	0.3

* *Not available*.

#### Weak population structure and low molecular relatedness in CartAP accessions

**Figure 6:**
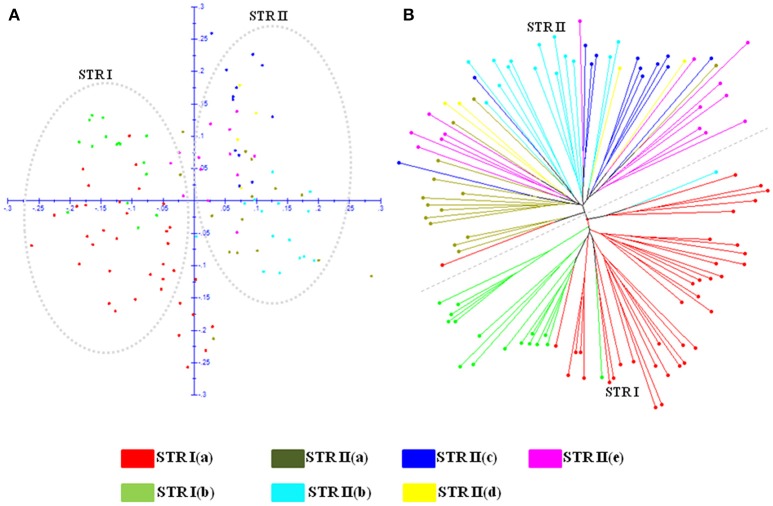
Demarcation of two major STRUCTURE populations (STR I and STR II) and seven sub-populations obtained by further hierarchical structure analysis on **(A)** PCoA scatter plot. **(B)** Neighbor Joining dendrogram. Color codes are provided for each sub-population.

Investigation of the population structure and genetic relatedness between accessions of an association panel is critical to circumvent spurious marker-trait associations (Yu and Buckler, 2006; Zhu et al., 2008; Yang et al., 2010; Nachimuthu et al., 2015). Structure analysis predicts the number of hypothetical sub-populations and identify admixed individuals among the studied panel while clustering analysis display the genetic relationships among accessions. In our study, we observed complete concordance between distance-based NJ and PCoA and Bayesian-based STRUCTURE analysis (Figures 6A,B). NJ clusters, NJ I and NJ II corresponded with STRUCTURE cluster STR I while NJ III coincided with STR II. Neither distance-based NJ analysis nor Bayesian-based STRUCTURE analysis could delimit the accessions on the basis of their geographic origin and/or distribution of studied traits. The lack of geographical structuring among CartAP accessions was expected since *Maximization* strategy was used for development of these core collections (Kumar et al., 2016), which emphasizes on maximization of the allelic diversity (both genetic and phenotypic) with minimum redundancy. For increasing representation of allelic diversity in the core collections, accessions with unique allelic combinations would have been selected and this might have assembled heterogeneous accessions from different regional pools resulting in an unstructured population. Additionally, an extensive number of genetic admixtures were identified with an ancestry share of < 80% in STRUCTURE analysis (Figures 3B,D). The presence of large number of admixed individuals can be attributed to incomplete lineage sorting during safflower diversification.

The range of *Fst*-value (0.0799–0.2888) suggests low to moderate differentiation between the seven sub-populations derived from hierarchical population structure analysis (Figure 4). At the population level, mean kinship coefficient (*Gij*) values (0–0.075) indicate low genetic similarity between these sub populations of the CartAP collection (Figure 5B). Additionally, high proportion of admixed individuals and the low pairwise kinship or co-ancestry values (*Fij*) at the individual level reflects no or weak relatedness between the safflower accessions (Figure 5A). The weak population structure coupled with low molecular relatedness between the sampled accessions of CartAP would prevent spurious marker-trait associations and confirms suitability of the CartAP panel for association mapping.

### Association mapping identifies significant marker-trait associations

Identification of loci influencing agronomic traits facilitates marker-assisted breeding to increase crop productivity. In safflower, efforts to identify molecular markers linked to traits of agronomic value are limited (Hamdan et al., 2008, 2012; Mayerhofer et al., 2010; García-Moreno et al., 2011; Pearl et al., 2014; Ebrahimi et al., 2017). In the present study, we performed association mapping and identified markers associated with eight traits of agronomic importance (seed oil content, oleic acid content, linoleic acid content, 100-seed weight, plant height, number of primary branches, days to 50% flowering and number of capitula per plant), many of which have been reported to influence crop yield (Patil, 1998). Of these, several marker-traits associations were found to be stable over the two growing seasons. Since MTAs are influenced by variations in environmental conditions, the strength of these associations needs to be analyzed through multi-location trials.

Low seed oil content is a serious impediment to adoption of safflower as a major oilseed crop globally. Oil content is a quantitative trait controlled by several genomic loci imparting small to moderate genetic effects, which are also influenced by environmental conditions (Hwang et al., 2014). Until now, no attempts have been made to map QTLs for oil content in safflower. Seed oil content in the CartAP accessions ranges from 16 to 50% (Table 6) indicating that the association panel includes diverse accessions which can be used in breeding programs. Our association study identified 11 MTAs correlated with oil content and supported by high correlation coefficient (*R*^2^ ≥ 10%; Table 7). Among these, two SSR loci, NGSaf_15 (*R*^2^ = 10.4–23.4%) and NGSaf_300 (*R*^2^ = 13.8–17.9%) were strongly associated with oil content in both GLM and MLM models and in both seasons (Table 7) and can be used to increase oil content of safflower cultivars.

The fatty acid composition of edible oil determines its utility and market value. Ever since the importance of oleic acid-rich dietary fats was elucidated, the demand for edible oil with high oleic acid content has increased substantially. Safflower is unique among oilseed crops due to the wide range of seed oil compositions available in its global germplasm (Velasco et al., 2005). Two major oil types are found in safflower—one with high linoleic acid and the other rich in oleic acid (Fernandez-Martinez et al., 1993). In addition, several lines with similar levels of linoleic and oleic acid have also been identified (Fernandez-Martinez et al., 1993). Modifying the fatty acid composition of safflower oil is particularly significant from the Indian perspective since majority of Indian cultivars are high in linoleic acid in spite of availability of high oleic lines in the Indian germplasm. Only one major QTL associated with oleic acid has been identified and it mapped on linkage group 3 using a cross between CL-1 (oleic acid: 14–22%) and CL-9 (oleic acid >84%; Hamdan et al., 2012). In addition to the major QTL associated with oleic acid, several modifying genes, which either enhance or repress the expression of major QTLs have been postulated to determine oleic acid content in safflower (Hamdan et al., 2008, 2012). In CartAP accessions, the oleic acid content ranged from 10 to 79% while linoleic acid varied from 13 to 87%. By association mapping, we identified three SSR loci (NGSaf_67, NGSaf_210 and NGSaf_309) linked with oleic acid content in safflower (Table 7). Among these loci, NGSaf_67 (*R*^2^ = 11.4–11.6%) was consistently associated with oleic acid content in both seasons and in both models while NGSaf_210 (displaying a phenotypic variation of ~14%) and NGSaf_309 (with the highest *R*^2^-value (~34%) among all the recorded marker-trait associations) were identified to be associated with oleic acid through MLM approach in both the seasons. We identified 10 significant marker-trait associations for linoleic acid content with *R*^2^ ≥ 10%. The NGSaf_210 locus (*R*^2^ = 14.3–17.9%) was associated with linoleic acid in both the models and seasons. In addition, NGSaf_67 (*R*^2^ = 10.9–16.7%) and NGSaf_309 (*R*^2^ = 34%) loci detected for oleic acid were also linked with linoleic acid content. Linoleic acid is synthesized from oleic acid by activity of fatty acid desaturase 2 enzyme encoded by the *FAD2* gene. A strong negative correlation has been reported between oleic acid and linoleic acid content in safflower (Kumar et al., 2016). Identification of common SSR loci (NGSaf_67, NGSaf_210 and NGSaf_309) for both these fatty acids indicates their strong linkage with the corresponding gene/s of the fatty acid biosynthetic pathway. These MTAs can serve as a useful resource for achieving the desired oil composition in the crop.

Plant height determines plant architecture and also influences crop yield. It is quantitatively inherited and a large number of QTLs associated with plant height have been reported in different crop systems (Wu et al., 2010; Morris et al., 2013; Zanke et al., 2014). In previous studies, safflower germplasm have revealed significant variations for plant height (Yeilaghi et al., 2015; Kumar et al., 2016). However, genetic loci influencing this trait have not yet been identified. The CartAP association panel harbors wide variation for plant height ranging from 94 to 226 cm. Association analysis identified 11 marker-trait associations with a *R*^2^ ≥ 10%. Two loci, NGSaf_156 (*R*^2^-value from 11.8 to 13.9%) and NGSaf_296 (*R*^2^-value from 12.7 to 14.7%) were associated with plant height in all environments except in the MLM model of 2011–2012 season. Plant height and flowering time are correlated traits as the onset of reproductive phase marks the termination of apical growth in safflower. Early maturing cultivars are desirable and several accessions with early flowering time were identified in CartAP (Kumar et al., 2016). Association analysis revealed that days to 50% flowering correlated significantly with 8 marker-trait associations which had *R*^2^ ≥ 10%. The NGSaf_92 locus (*R*^2^ = 7.9–11.6%) was associated with days to 50% flowering for all models and seasons except GLM of 2011–2012 season. Interestingly, we detected two markers (NGSaf_101 and NGSaf_296), which were associated with both plant height and days to 50% flowering in either one of the models or seasons (Table 7). Plant height and flowering time are traits that are highly influenced by environmental effects. Therefore, seasonal variations between the two growing periods could have led to differences in detection of these MTAs in the studied seasons and models. Nevertheless, these common MTAs provide an initial platform to study the genetic relationships between the two traits.

The number of primary branches also defines plant architecture and influences yield since primary branches produce secondary to quaternary branches, each of which terminates into the characteristic globular head of safflower. Association analysis for number of primary branches detected 6 marker-trait associations, which showed correlation coefficient (*R*^2^-value) ≥ 10%. The locus NGSaf_279 (*R*^2^-value ranging from 8 to 10.8%) was associated with primary branches using both the models in both growing seasons. The number of capitula in accessions of the CartAP collection ranged from 16 to 203 (Kumar et al., 2016). The number of capitula per plant exhibited 2 significant associations which had *R*^2^ ≥ 10%. The NGSaf_279 locus (*R*^2^ = 7.4–9.3%) was common among all models and for both seasons. A positive correlation was reported by Kumar et al. (2016) between the number of capitula per plant and number of primary branches, which is in congruence with our results on identification of the SSR locus NGSaf_279, as a common marker for both the traits.

A mature safflower achene consists of 33–60% hull and 40–67% kernel (Dajue and Mündel, 1996). It has been suggested that reducing hull content can significantly enhance seed oil content in safflower (Dajue and Mündel, 1996). Association analysis of 100-seed weight among CartAP accessions identified 7 significant marker-trait associations with *R*^2^ ≥ 10%. With the exception of season 2012–2013 MLM approach, two loci NGSaf_306 (*R*^2^ = 13–24.5%) and NGSaf_309 (*R*^2^ = 15.5–24.1%), were consistently associated with 100-seed weight (Table 7). These loci may play an important role in deciphering the molecular relationship between hull thickness and oil content.

Although safflower is an important oilseed crop with global distribution, there have been limited attempts to identify marker-trait associations for crop improvement. The present study established the suitability of CartAP collection for association mapping due to the absence of population structure and presence of significant genetic and phenotypic diversity. Using association mapping we were able to identify significant marker-trait associations for eight important agronomic traits. Many of these associations were consistently detected over two growing seasons and multiple models. The stability and utility of these marker-trait associations need to be analyzed further under additional environments through multi-location trials. The potential marker-trait associations identified in this study will be useful in facilitating marker-assisted breeding for crop improvement and identification of candidate genes for trait variability in safflower.

## Author contributions

SG and AJ conceived and designed the experiments; HA and SK conducted the experiments, collected and analyzed the data; HA, SK, AJ, and SG wrote the manuscript; MA, AK, AJ, and SG provided facilities for completion of experiments and reviewed the manuscript.

### Conflict of interest statement

The authors declare that the research was conducted in the absence of any commercial or financial relationships that could be construed as a potential conflict of interest.

## Footnotes

^1^www.fao.org/faostat/en/#data/QD
