# Supplementary material for: Association Mapping for Important Agronomic Traits in Safflower (Carthamus tinctorius L.) Core Collection Using Microsatellite Markers
Source: Front Plant Sci. 2018 Mar 29;9:402. doi: 10.3389/fpls.2018.00402 (PMC5885069; doi:10.3389/fpls.2018.00402)
Supplement: Supplementary file 1 [file Table1.PDF]

Supplementary Table 1. Plant material used in the present study

S.No.	PI number	Country of origin	Core Collection[#]
1	514619	China	CartC1/CartC2
2	543977	China	CartC1/CartC2
3	544007	China	CartC1/CartC2
4	544031	China	CartC1
5	544040	China	CartC1/CartC2
6	544043	China	CartC1/CartC2
7	544055	China	CartC1/CartC2
8	401473	Bangladesh	CartC1/CartC2
9	401477	Bangladesh	CartC1/CartC2
10	401478	Bangladesh	CartC1/CartC2
11	401479	Bangladesh	CartC1/CartC2
12	470942	Bangladesh	CartC1/CartC2
13	305193	India	CartC1
14	283764	India	CartC1
15	305204	India	CartC1/CartC2
16	306825	India	CartC1/CartC2
17	306892	India	CartC1
18	306912	India	CartC1/CartC2
19	307001	India	CartC1/CartC2
20	401577	India	CartC1/CartC2
21	401589	India	CartC1
22	388906	Iran	CartC1/CartC2
23	405968	Iran	CartC1/CartC2
24	388902	Iran	CartC1/CartC2
25	198990	Israel	CartC1
26	306684	Israel	CartC1/CartC2
27	386173	Syria	CartC1/CartC2
28	340076	Turkey	CartC1
29	407617	Turkey	CartC1
30	306597	Egypt	CartC1/CartC2
31	306594	Egypt	CartC1/CartC2
32	250081	Egypt	CartC1
33	306610	Egypt	CartC1/CartC2
34	193475	Ethiopia	CartC1/CartC2
35	253560	Morocco	CartC1/CartC2
36	576990	Czechoslovakia	CartC1
37	576985	France	CartC1/CartC2
38	253522	Italy	CartC1/CartC2
39	258421	Portugal	CartC1
40	262442	Spain	CartC1/CartC2
41	369845	Tajikistan	CartC1/CartC2
42	537607	USA	CartC1/CartC2
43	560171	USA	CartC1/CartC2

S.No.	PI number	Country of origin	Core Collection[#]
44	537619	USA	CartC1/CartC2
45	537635	USA	CartC1/CartC2
46	537653	USA	CartC1/CartC2
47	537656	USA	CartC1
48	537658	USA	CartC1/CartC2
49	537663	USA	CartC1/CartC2
50	537674	USA	CartC1/CartC2
51	537706	USA	CartC1/CartC2
52	560168	USA	CartC1/CartC2
53	560169	USA	CartC1/CartC2
54	560172	USA	CartC1
55	560181	USA	CartC1
56	242419	Australia	CartC1/CartC2
57	369849	Russia	CartC1/CartC2
58	514618	China	CartC2
60	514622	China	CartC2
61	543981	China	CartC2
62	543993	China	CartC2
63	544011	China	CartC2
64	544029	China	CartC2
65	544033	China	CartC2
66	543974	China	CartC2
67	401476	Bangladesh	CartC2
68	401470	Bangladesh	CartC2
69	305218	India	CartC2
70	306972	India	CartC2
71	306836	India	CartC2
72	306866	India	CartC2
73	306876	India	CartC2
74	306897	India	CartC2
75	306906	India	CartC2
76	306915	India	CartC2
77	306944	India	CartC2
78	306993	India	CartC2
79	307030	India	CartC2
80	307067	India	CartC2
81	374222	Pakistan	CartC2
82	304408	Pakistan	CartC2
83	268374	Afghanistan	CartC2
84	250709	Iran	CartC2
85	392026	Turkey	CartC2
86	304442	Iran	CartC2
87	343777	Iran	CartC2
88	405960	Iran	CartC2
89	405970	Iran	CartC2

S.No.	PI number	Country of origin	Core Collection[#]
90	405987	Iran	CartC2
91	251262	Jordan	CartC2
92	253895	Syria	CartC2
93	340079	Turkey	CartC2
94	304503	Turkey	CartC2
95	340081	Turkey	CartC2
96	306593	Egypt	CartC2
97	305527	Sudan	CartC2
98	209300	Kenya	CartC2
99	273876	Eritrea	CartC2
100	253515	Germany	CartC2
101	305537	Uzbekistan	CartC2
102	348915	Canada	CartC2
103	537111	Mexico	CartC2
104	537614	USA	CartC2
105	560175	USA	CartC2
106	537634	USA	CartC2
107	537641	USA	CartC2
108	537659	USA	CartC2
109	537671	USA	CartC2
110	537681	USA	CartC2
111	537682	USA	CartC2
112	537701	USA	CartC2
113	537707	USA	CartC2
114	537710	USA	CartC2
115	560178	USA	CartC2
116	560183	USA	CartC2
117	209289	Unknown origin	CartC2
118	307114	India	CartC2
119	426186	Afghanistan	CartC1/CartC2
120	Annigeri	India	Indian cultivar
121	Manjira	India	Indian cultivar
122	Sharda	India	Indian cultivar
123	PBNS-12	India	Indian cultivar
124	TSF-1	India	Indian cultivar

[#]CartC1 and CartC2 refers to composite core collections reported by Kumar et al., 2016.
