# Supplementary material for: Association Mapping for Important Agronomic Traits in Safflower (Carthamus tinctorius L.) Core Collection Using Microsatellite Markers
Source: Front Plant Sci. 2018 Mar 29;9:402. doi: 10.3389/fpls.2018.00402 (PMC5885069; doi:10.3389/fpls.2018.00402)
Supplement: Supplementary file 2 [file Table2.PDF]

Supplementary Table 2. Climatic data including temperature variation (Range and Average) and humidity (Average) for two growing seasons (2011-2012 and 2012-2013).

Month	Season 2011-2012		Season 2012-2013	
	Temperature Min -Max (Avg)# (°C)	Humidity Avg# (%)	Temperature Min -Max (Avg) # (°C)	Humidity Avg# (%)
**October**	23-35 (29)	65	24-35 (29)	63
**November**	18-32 (24)	72	17-30 (23)	78
**December**	15-28 (21)	74	15-26 (19)	72
**January**	13-24 (18)	75	15-24 (17)	77
**February**	16-30 (23)	56	17-26 (21)	77
**March**	22-36 (28)	52	22-34 (28)	63
**April**	26-38 (32)	48	27-40 (33)	43

# Min- Minimum, Max- Maximum, Avg- Average

Note: Average precipitation for both the two growing seasons was 0mm.

Source: https://www.wunderground.com
