# Supplementary material for: Association Mapping for Important Agronomic Traits in Safflower (Carthamus tinctorius L.) Core Collection Using Microsatellite Markers
Source: Front Plant Sci. 2018 Mar 29;9:402. doi: 10.3389/fpls.2018.00402 (PMC5885069; doi:10.3389/fpls.2018.00402)
Supplement: Supplementary file 3 [file Table3.PDF]

Supplementary Table 3. Marker statistics and linkage group distribution of each SSR locus.

SSR locus	Na	M_{AF}	*Ho*	*He*	PIC	HWE	LG
**NGSaf_2**	4	0.770	0.053	0.385	0.357	*	9
**NGSaf_9**	2	0.650	0.034	0.455	0.352	*	-
**NGSaf_12**	3	0.688	0.075	0.435	0.347	*	12
**NGSaf_13**	2	0.914	0.008	0.157	0.145	*	4
**NGSaf_14**	2	0.951	0.000	0.094	0.089	*	5
**NGSaf_15**	3	0.788	0.083	0.340	0.290	*	3
**NGSaf_20**	3	0.586	0.121	0.502	0.396	*	-
**NGSaf_22**	3	0.522	0.026	0.544	0.443	*	4
**NGSaf_23**	3	0.786	0.076	0.339	0.284	*	6
**NGSaf_28**	2	0.635	0.033	0.455	0.342	*	4
**NGSaf_34**	2	0.564	0.010	0.492	0.371	*	-
**NGSaf_39**	2	0.626	0.009	0.468	0.359	*	4
**NGSaf_43**	2	0.920	0.008	0.147	0.136	*	6
**NGSaf_44**	2	0.618	0.079	0.472	0.361	*	2
**NGSaf_45**	2	0.531	0.000	0.498	0.374	*	-
**NGSaf_48**	2	0.806	0.019	0.313	0.264	*	-
**NGSaf_49**	3	0.877	0.082	0.222	0.209	*	4
**NGSaf_56**	2	0.651	0.025	0.454	0.351	*	-
**NGSaf_63**	2	0.664	0.000	0.446	0.347	*	-
**NGSaf_65**	3	0.768	0.018	0.371	0.323	*	-
**NGSaf_67**	4	0.748	0.035	0.391	0.335	*	7
**NGSaf_69**	5	0.450	0.028	0.709	0.669	*	-
**NGSaf_73**	3	0.763	0.080	0.365	0.305	*	6
**NGSaf_83**	3	0.419	0.381	0.626	0.547	*	3
**NGSaf_84**	2	0.810	0.034	0.307	0.260	*	5
**NGSaf_89**	4	0.711	0.029	0.422	0.348	*	8
**NGSaf_91**	2	0.868	0.033	0.229	0.203	*	-
**NGSaf_92**	3	0.799	0.123	0.324	0.277	*	9
**NGSaf_94**	3	0.654	0.058	0.458	0.361	*	11
**NGSaf_98**	2	0.509	0.000	0.500	0.375	*	10
**NGSaf_101**	4	0.725	0.090	0.439	0.402	*	4
**NGSaf_105**	2	0.954	0.025	0.087	0.084	*	-
**NGSaf_111**	6	0.517	0.125	0.554	0.458	*	2
**NGSaf_114**	2	0.573	0.000	0.489	0.370	*	5
**NGSaf_115**	3	0.682	0.008	0.448	0.368	*	3
**NGSaf_117**	2	0.992	0.000	0.016	0.016	*	4
**NGSaf_130**	2	0.571	0.000	0.490	0.370	*	-
**NGSaf_138**	2	0.971	0.025	0.056	0.055	*	9
**NGSaf_142**	2	0.585	0.026	0.485	0.368	*	-

**NGSaf_145**	2	0.790	0.000	0.331	0.276	*	6
**NGSaf_148**	2	0.685	0.129	0.431	0.338	*	7
**NGSaf_151**	2	0.530	0.000	0.498	0.374	*	3
**NGSaf_152**	4	0.388	0.463	0.709	0.657	*	8
**NGSaf_154**	4	0.516	0.000	0.530	0.421	*	12
**NGSaf_155**	4	0.342	0.132	0.718	0.666	*	10
**NGSaf_156**	5	0.636	0.079	0.492	0.408	*	6
**NGSaf_158**	3	0.870	0.056	0.227	0.203	*	-
**NGSaf_164**	3	0.769	0.034	0.371	0.327	*	-
**NGSaf_173**	2	0.504	0.000	0.500	0.375	*	5
**NGSaf_178**	3	0.579	0.075	0.494	0.380	*	1
**NGSaf_181**	4	0.826	0.083	0.302	0.282	*	12
**NGSaf_201**	4	0.546	0.092	0.603	0.540	*	11
**NGSaf_204**	2	0.938	0.008	0.116	0.110	*	11
**NGSaf_210**	3	0.648	0.400	0.518	0.463	*	4
**NGSaf_211**	2	0.517	0.109	0.499	0.375	*	3
**NGSaf_236**	4	0.511	0.103	0.643	0.589	*	-
**NGSaf_237**	2	0.521	0.843	0.499	0.375	*	-
**NGSaf_238**	3	0.702	0.042	0.444	0.380	*	-
**NGSaf_239**	2	0.719	0.188	0.404	0.323	*	-
**NGSaf_242**	6	0.426	0.157	0.663	0.603	*	-
**NGSaf_245**	3	0.700	0.018	0.465	0.419	*	-
**NGSaf_248**	2	0.951	0.000	0.094	0.089	*	-
**NGSaf_255**	5	0.545	0.125	0.523	0.416	*	1
**NGSaf_257**	6	0.616	0.388	0.564	0.520	*	-
**NGSaf_259**	4	0.771	0.092	0.368	0.323	*	-
**NGSaf_261**	4	0.627	0.123	0.485	0.389	*	-
**NGSaf_262**	3	0.664	0.035	0.452	0.358	*	-
**NGSaf_264**	6	0.563	0.092	0.627	0.589	*	-
**NGSaf_265**	8	0.711	0.116	0.467	0.438	*	-
**NGSaf_266**	3	0.899	0.017	0.186	0.178	*	-
**NGSaf_273**	3	0.748	0.034	0.379	0.310	*	-
**NGSaf_276**	2	0.789	0.333	0.031	0.277	*	-
**NGSaf_279**	3	0.727	0.050	0.434	0.393	*	6
**NGSaf_281**	7	0.393	0.033	0.763	0.734	*	5
**NGSaf_282**	8	0.603	0.143	0.592	0.558	*	-
**NGSaf_286**	7	0.429	0.124	0.697	0.650	*	-
**NGSaf_289**	3	0.662	0.475	0.050	0.398	*	-
**NGSaf_292**	2	0.800	0.033	0.320	0.269	*	-
**NGSaf_294**	4	0.454	0.958	0.615	0.538	*	-
**NGSaf_295**	4	0.750	0.086	0.415	0.389	*	-
**NGSaf_296**	5	0.632	0.058	0.550	0.508	*	6

**NGSaf_300**	6	0.705	0.590	0.477	0.452	NS	12
**NGSaf_301**	4	0.642	0.160	0.482	0.394	*	6
**NGSaf_306**	4	0.506	0.205	0.549	0.449	*	6
**NGSaf_307**	3	0.719	0.082	0.439	0.394	*	-
**NGSaf_308**	4	0.521	0.442	0.622	0.562	*	-
**NGSaf_309**	5	0.465	0.115	0.649	0.585	*	5
**NGSaf_310**	3	0.417	0.124	0.651	0.576	*	-
**NGSaf_313**	3	0.703	0.119	0.459	0.412	*	-
**NGSaf_314**	4	0.510	0.038	0.632	0.572	*	-
**NGSaf_322**	3	0.517	0.067	0.577	0.490	*	-
**NGSaf_323**	4	0.692	0.152	0.480	0.439	*	-
**NGSaf_324**	5	0.380	0.132	0.679	0.617	*	-
**Mean**	3.344	0.660	0.112	0.438	0.381	*	-

Na- Number of alleles, M_{AF}- Major allele frequency, *Ho*- Observed heterozygosity, *He*- Expected heterozygosity, PIC- Polymorphic information content, HWE- Hardy-Weinberg equilibrium ($P < 0.05$); * indicates significant deviation from HWE and NS refers to non-significant, LG- Linkage groups identified based on Bowers et al. (2016).
